# How does mathematics anxiety impair mathematical abilities? Investigating the link between math anxiety, working memory, and number processing

**DOI:** 10.1371/journal.pone.0211283

**Published:** 2019-01-25

**Authors:** Kenny Skagerlund, Rickard Östergren, Daniel Västfjäll, Ulf Träff

**Affiliations:** 1 Department of Behavioural Sciences and Learning, Linköping University, Linköping, Sweden; 2 JEDILab, Division of Economics, Department of Management and Engineering, Linköping University, Linköping, Sweden; French National Center for Scientific Research (CNRS) & University of Lyon, FRANCE

## Abstract

In contemporary society, it is essential to have adequate mathematical skills. Being numerate has been linked to positive life outcomes and well-being in adults. It is also acknowledged that math anxiety (MA) hampers mathematical skills increasingly with age. Still, the mechanisms by which MA affect performance remain debated. Using structural equation modeling (SEM), we contrast the different ways in which MA has been suggested to interfere with math abilities. Our models indicate that MA may affect math performance through three pathways: (1) indirectly through working memory ability, giving support for the ‘affective drop’ hypothesis of MA’s role in mathematical performance, (2) indirectly through symbolic number processing, corroborating the notion of domain-specific mechanisms pertaining to number, and (3) a direct effect of MA on math performance. Importantly, the pathways vary in terms of their relative strength depending on what type of mathematical problems are being solved. These findings shed light on the mechanisms by which MA may interfere with mathematical performance.

## Introduction

Learning mathematics is a complex endeavor that is both cognitively and, sometimes, emotionally challenging. Still, in contemporary society, it is essential to have adequate mathematical skills. Lack thereof can severely hamper one’s prospects of making well-informed decisions about financial matters and other aspects relating to one’s psychological and physical well-being [1] [2] [3]. Decisions relying on numerical abilities are ubiquitous in every aspect of life, ranging from trivial everyday interactions in the local supermarket to significant choices about whether to buy a house, switching careers, and whether to undergo risky medical treatments [4]. Therefore, being able to understand and use numerical information is imperative, both from the perspective of the single individual, but also for society as a whole. However, far from everyone is functionally numerate and it is estimated that roughly 25% of the British population suffers from low numeracy [5]. In turn, low numeracy in the population constitutes a major socio-economical cost to nations [1]. In the American population, a large nationwide survey indicates that roughly half of the adult population lack the minimal numerical skills required to use numbers in printed materials, such as calculating change in price menus [6]. Thus, it is absolutely essential to investigate how we can foster a fertile learning environment in the early school years and also investigate how these mathematical and cognitive abilities develop into adulthood. One important emotional factor that adversely affects individuals’ prospects of attaining adequate math skills is mathematics anxiety (MA), which can be defined as “…feelings of tension and anxiety that interfere with the manipulation of numbers and the solving of mathematical problems in a wide variety of ordinary life and academic situations.” [7]. The detrimental effect of MA on mathematical performance is well-established (see [8] for a review), but the exact mechanisms by which it hampers performance remain inconclusive. The prevalence rate has been reported to be between around 11% in university students [7] and 17% in the population [9]. This high frequency is alarming given the negative impact of MA on math ability. Researchers have proposed that MA may develop as a result of a sense of failure in math, negative attitude transfer from teachers, or due to cognitive factors [10]. Some findings indicate that MA hampers attentional resources and working memory (WM) processes that in turn impedes mathematical operations (e.g., [11]), whereas others argue that MA undermines more basic number processing abilities (e.g., [12] [10]). While researchers have made significant progress in trying to understand how mathematical abilities develop throughout ontogeny (e.g., [13] [14] [15] [16]) in terms of the cognitive abilities that underlie typical and atypical achievement, surprisingly little is known about the manifestation of how MA affects math performance.

The focus of the current study is to contribute to our understanding of the mechanisms by which MA undermines mathematical abilities in adults. Specifically, we will juxtapose the different hypotheses, briefly mentioned above, that have been proposed in previous research. Measuring mathematical abilities in a comprehensive manner *in situ* is always challenging given the multi-faceted nature of mathematics. Importantly, research has shown that different cognitive abilities support different aspects of mathematics [16] [17] [18] [19] and it is not clear whether MA undermines all aspects of math to the same degree. Acknowledging this, we investigate two different aspects of mathematical abilities in adults: (1) *numeracy*, and (2) *arithmetic calculation*. Recent advances have been made in trying to define and operationalize the notion of numeracy in adults more systematically [20] [21] [4] [6] [22]. Numeracy denotes the basic understanding of the number line, time, measurement, and estimation, as well as higher level concepts such as fractions, proportions, percentages, and probabilities [21]. Numeracy has been tied to the ability to assess risk in the medical domain, where more numerate women made more accurate assessments of the risks involved in undergoing mammography [22]. Numeracy has also been linked to more normative decision-making in general and less susceptibility to cognitive biases such as the *framing effect* (e.g., [4] [6]). The Berlin Numeracy Test (BNT; [20]) and the numeracy test developed by Weller and colleagues [23] are examples of instruments that have been used to measure adult numeracy. The BNT is supposed to measure overall numeracy that is predictive of decision-making skills, but it remains unknown whether and how MA affects numeracy. The BNT measures something else than arithmetic calculation skills insofar as it measures basic understanding of probabilities and text-based problem solving skills whereas arithmetic calculation is thoroughly about numerical operations. Here, we use structural equation modeling (SEM) to investigate whether MA relates to both numeracy and arithmetic through the same pathways. Juxtaposing these aspects may explain the disparity in the previous literature regarding the role of MA and enhance our understanding of the mechanisms involved.

The literature overview provided below is divided into three sections. The first section concerns WM ability in the relation to mathematical abilities, the second concerns the link between basic number processing skills and mathematical abilities, and the final section concerns the relation between MA and mathematical ability.

### Working memory ability and mathematics

Mounting evidence suggests that WM ability is involved in mathematical reasoning. However, these studies have primarily focused on disentangling the role of different WM components in childhood. Still, the cognitive abilities that have been identified as being important predictors throughout the school years are likely influential for mathematics in later adulthood. Abundant research has shown that WM (e.g., [24] [25] [26]) and semantic long-term memory (e.g., [27] [28]), are important cognitive abilities involved during mathematical performance. Working memory is believed to provide a flexible and efficient mental workspace that is involved in handling the storage and updating of task-relevant information involved in complex arithmetic tasks [28] [18] [29]. Working memory ability has been associated with mathematical ability in early childhood (e.g., [30]) as well as in older children (e.g., [31]) and adolescents (e.g., [32]). Nevertheless, there is a disparity concerning which of the WM components that are linked to mathematical ability. Some researchers have found that the tasks tapping the phonological loop is predictive of ability (e.g., [33]), while others have found that visuospatial WM ability is a more potent predictor (e.g., [34]). This disparity has led to the suggestion that there is a developmental shift in reliance on WM components (cf. [35]), where children in 2^nd^ grade move from relying on phonological abilities to increasingly rely on visuospatial abilities. Still, WM ability as a whole is arguably an influential cognitive component involved in mathematical ability throughout ontogenetic development and into adulthood.

### Number processing skills and the relation to mathematics ability

The notion of a number sense [36] is firmly established in the research literature and refers to the finding that human beings are endowed with an innate domain-specific ability to represent and manipulate quantities [36]. This is an ability phylogenetically shared with other species and is believed to provide a foundation for the subsequent acquisition of the culturally derived symbolic system and, ultimately, mathematics [36] [37]. Both symbolic and non-symbolic abilities have been shown to play a crucial role in mathematics achievement [38] [16] [39]. Together with the basic capacity to understand and represent non-symbolic quantities, the ability to associate the quantities with symbolic referents is arguably the rudimentary building block of mathematics. The affinity with symbolic numbers is often measured using digit comparison paradigms in which one is asked to quickly estimate which of two simultaneously presented numerals is the largest (e.g., “5 vs. “8”). In studies of children, there is a strong relationship between mathematics ability and response times on digit comparison tasks, which indicate that they have mastered the number line and have quick access to the underlying semantic representations [40]. Symbolic number processing ability has been associated with adult mathematical ability measured using an arithmetic test [41]. What exactly is measured using a number discrimination task? A recent study made a rigorous examination of this type of task to tease out what the processes involved are and how this task is related to mathematics abilities in adults [42]. The authors concluded that number discrimination requires processing of the connection between the Arabic symbols and the underlying referents, as argued previously by others, but also the ordinal connection between symbols as well. The ordinal processing may involve associative chaining mechanisms in long-term memory, which in turn links to more complex mathematical skills [42]. Thus, although inconclusive, there is empirical support for the notion that basic number processing abilities continues to be important in adults and not only in children. In addition, some researchers have argued that MA hampers this very system.

### Mathematics anxiety and the relation to mathematics ability

Mathematics anxiety is related to poor math performance and indirectly to education and career path choice [11] [43] [44]. Findings indicate that MA persists into adulthood because of avoidance behavior of math courses and engagement in daily activities and decisions that require arithmetic [44]. Still, the origins and development of MA and exactly how it affects mathematics ability and learning outcomes is still debated. In terms of cognitive mechanisms that may underlie MA, researchers have suggested that feelings of anxiety during math calculations takes up WM resources that in turn impedes performance [11] [9] [45]. This *affective drop* [9] may be driven by self-referential negative thoughts and feelings in the moment of doing arithmetic computations that hampers efficient use of limited WM resources. Along these lines, a finding from cognitive neuroscience suggests a plausible neurocognitive mechanism of how MA interferes with domain-general cognitive processing. Pletzer and colleagues [46] conducted an fMRI study using two groups of adults with low or high MA. The authors found that MA leads to ineffective deactivation of the default mode network (DMN) in the brain. The DMN is largely engaged in self-referential and emotional processing when there are no immediate demands on the central executive network that is engaged during goal-directed and effortful processing [47]. The insula, together with the anterior cingulate cortex (ACC), comprises a salience network [47] that is responsible for the detection of environmentally salient stimuli. The salience network regulates the deactivation of the DMN and the activation of the central executive network (CEN) as a response to salient events that require attention [47] [48]. This would explain why MA affects mathematical abilities through interference of WM processes. Additional neuroimaging data point to the fact that individuals with MA show significant activity in the insula and mid cingulate cortex (two nodes involved in the pain network of the brain) as a response to anticipating upcoming math tasks [49]. Synthesizing the results from the aforementioned studies, one might hypothesize that the aberrant activity of insula (i.e., as a pain response) hampers the deactivation of the DMN and the engagement of the CEN in individuals with MA. In children, Young, Wu and Menon [50] demonstrated that MA was associated with lower activity in brain areas subserving WM and attention, such as the dorsolateral prefrontal cortex, and areas supporting numerical processing, such as the parietal cortex. The children with MA instead showed heightened neural responses in the amygdala, which indicates that they show increased processing of negative emotions. These neuroimaging studies reported above point to various potential pathways in which MA may interfere with mathematical processing: (a) Numerical processing may be hampered through dysfunctional neurocognitive activation patterns in the parietal cortex, which is involved in non-symbolic number processing as well as symbolic number processing and arithmetic, and (b) through aberrant activity in the DMN, insula, and amygdala that impedes domain-general cognitive abilities, such as WM. In line with the former, recent research has suggested that basic numerical abilities may be affected (e.g, [51] [10] [12]). The study by Maloney and colleagues [12] found that adults with high MA showed poorer performance on a simple symbolic number processing task than adults with low MA. This is interesting given that this type of task is devoid of demands on WM resources, which would go against the notion that MA primarily affects math performance by evoking negative feelings that distracts from the taxing task at hand. Still, others have found that the effect of MA on performance seem to be proportional to the complexity of the mathematical task at hand [11] [8], which may indicate that MA works through multiple pathways: (1) a pathway through WM in which WM capacity is diminished as a result of emotional and cognitive control demands, and (2) a more basic number processing pathway in which processing of numerical stimuli is affected. In addition, given that individuals suffering from MA may avoid math courses and engagement in daily activities and decisions that require arithmetic, as suggested by Hembree [44], it is also plausible that MA affects math ability in a more temporally distributed and distal way. Thus, it is likely that MA affect math ability as an avoidance effect in conjunction with more proximal cognitive effects (i.e. through WM or basic number processing). Therefore, we wished to investigate the role of MA by utilizing a sample that allows us to incorporate measures of WM abilities and basic number processing to get a more nuanced view of the role of math anxiety in adult math performance. Studies investigating the link between MA and math ability using a comprehensive test battery of both basic number processing and general cognitive abilities are scarce. A very recent contribution comes from Douglas and Lefevre [52] who investigated the influence of cognitive abilities and basic number skills on MA using SEM. They found that there was no direct link between neither cognitive abilities nor basic number skills to MA. Instead, the authors found that complex math performance fully mediated the relations between basic cognitive skills and MA. In addition, there was no direct link between basic number processing and MA [52]. The link between MA and math abilities is likely complex and, as Douglas and Lefevre [52] noted, there are likely several factors that may be at play. Nevertheless, using SEM to include several predictors in a structural model is a fruitful way of illuminating the relationship between these intricate variables.

In sum, results are inconclusive regarding how MA interferes with mathematical ability in adults. Except for Douglas and Lefevre [52], no study has explicitly juxtaposed different hypotheses of how MA is believed to hamper mathematical computations while including WM abilities and number processing skills in the models using SEM. This is what we sought to address.

### Purpose of the current study

The overarching goal of the current study was to try to disentangle the mechanisms by which MA may operate and interfere with mathematical abilities in adults. We collected data from a sample of adults (*N* = 170). The upside of using SEM rather than, say, multiple regression analysis is that we can investigate the model as a whole and investigate direct and indirect effects. Also, given that we have prior theoretical support for the relative involvement of the psychological constructs, it allows for confirmatory testing of a complete model [53]. This allows us to address the following questions:

1. Does MA interfere with mathematical ability by affecting WM processes?
2. Does MA impair mathematical ability through a weakening of basic number processing skills?
3. Does MA have a distal effect on math ability as an avoidance effect?
4. Is the underlying mechanism by which MA hampers math ability the same regardless of the type of mathematical task?

### Model prediction and hypotheses

In the current study, we hypothesize that number processing should contribute to both arithmetic and numeracy, the latter given that numeracy supposedly taps the ability to process ratios, fractions, probabilities and percentages. We will investigate whether we can model number processing as a latent factor consisting of one- and two-digit symbolic number processing as indicators, which in turn predicts numeracy and arithmetic.

**Fig 1:**
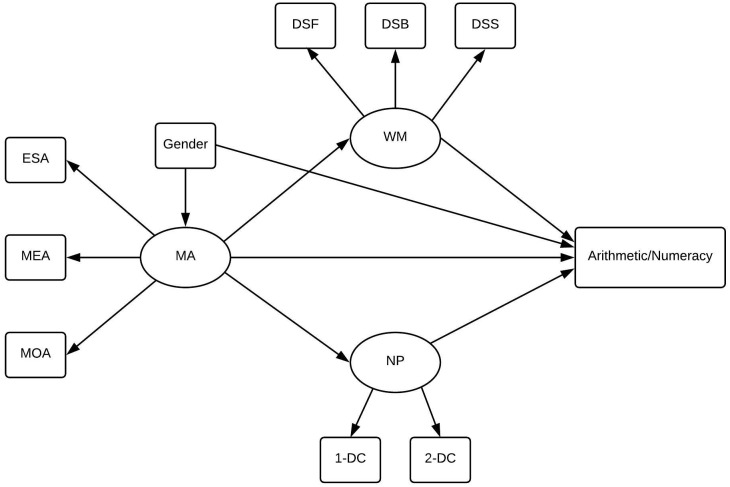
Conceptual model. Hypothesized conceptual model of pathways between math anxiety (MA), working memory (WM), number processing (NP) and their relation to numeracy and arithmetic.

We also hypothesize that we can model WM as a latent factor, consisting of subtests of digit span from Wechsler Adult Intelligence Scale IV (WAIS-IV, [54]) that subsequently is involved in both numeracy and arithmetic. In terms of MA, we investigate different contrasting hypotheses regarding the direction of influence, thus being illustrated in Fig 1 as having both direct and indirect effects to be tested. We test whether MA affects mathematics performance through number processing (cf. [51]) or through WM (cf. [11]). See Fig 1 for a conceptual model containing the hypothesized paths between variables.

## Method

### Participants

The sample consisted of 170 Swedish adults (85 men and 85 women, mean age = 24.06, *SD* = 3.39) who were students at Linköping University. The participants were recruited from different academic disciplines and years into their education. All participants had normal or corrected-to-normal vision and normal color vision. We excluded participants with a history of neurologically based impairments, such as ADHD or other known learning disabilities (e.g., dyslexia and dyscalculia). All participants gave their informed and written consent and the study was approved by the regional ethics committee in Linköping, Sweden.

### Measure of WM

Working memory ability was assessed using the digit span subtest of WAIS-IV. This subtest is divided into three conditions: Digit Span Forward (DSF), Digit Span Backward (DSB), and Digit Span Sequencing (DSS). In the first condition, the participant hears a series of digits and attempts to repeat them out loud in order. In contrast, in the Digit Span Backward condition the participant has to repeat the string of digits in reverse order. The sequencing condition requires the participant to recall all the digits in correct ordinal sequence. All conditions become increasingly more difficult in terms of the number of digits there are to be repeated. The maximum score for each condition is 16 for a total 48 for the entire task. Cronbach’s alpha was calculated by checking the internal consistency across each subtest of WAIS-IV and resulted in α = .71.

### Measures of basic number processing

Symbolic number processing was measured using both one-digit comparison and two-digit comparison conditions. The former consisted of two Arabic one-digit numerals ranging from 1–9 that were simultaneously and horizontally displayed on a computer screen. The objective in this task was to decide which of the two numerals was the numerically larger one, and respond with either “A”, corresponding to the left numeral, or “*” corresponding to the right-most numeral. Before each trial, a fixation cross was displayed for 1000 ms, after which two digits were presented and remained exposed to the participant until he/she pressed a button. Two numerical distances were used: 1 (e.g., “3–4”) and 4–5 (e.g., “2–7” and “1–5”), and each pair was presented twice resulting in a total of 32 trials. The response times and errors were registered for each trial by the software program, and only response times for correct responses were recorded and used in the analysis. The mean response time for each participant was used as the dependent variable. The two-digit comparison task (2-DC) involved the same general setup as the one-digit condition (1-DC), and mean response time was used as the dependent measure. Cronbach’s alpha was calculated by checking the internal consistency of the reaction times across both the 1-DC and the 2-DC and resulted in α = .83.

### Measure of math anxiety

Emotional attitude towards mathematics and numbers was assessed using the *Mathematics Anxiety Scale-UK* (MAS-UK; [55]). The MAS-UK is a questionnaire containing 23 statements concerning varying situations such as *“I feel worried when working out how much change a cashier should have given me in a shop after buying several items*.*“*. The respondent then indicates on a Likert type scale from 1 (“*Not at all”*) to 5 (“*Very much”*) how worried they feel in the corresponding situation. The items load on three different factors: Everyday/Social Math Anxiety (ESA), Math Observation Anxiety (MOA), and Math Evaluation Anxiety (MEA). Cronbach’s alpha was calculated by checking the internal consistency across each factor of MAS-UK and resulted in α = .70.

### Measures of mathematics ability

#### Numeracy

Numeracy was measured using the Berlin Numeracy Test (BNT), developed by Cokely et al. [20] and validated in Swedish by Lindskog, Kerimi, Winman, and Juslin [56]. This scale was chosen since it has proven to be normally distributed in an educated population and has shown good discriminant and convergent validity with other cognitive tests [20]. The BNT consists of four items (e.g., “*Imagine we are throwing a five-sided die 50 times*. *On average*, *out of these 50 throws how many times would this five-sided die show an odd number (1*, *3 or 5)*?). The BNT can be administered in an adaptive format, which is less time consuming and requires that the participant only completes three problems. However, we chose to use all four items of the scale and sum up all correct answers as an index of numeracy, which is a procedure suggested as a valid alternative by Cokely and colleagues [20]. The participants had 10 minutes at their disposal to solve all four items of the BNT. Cronbach’s alpha was calculated by checking the internal consistency across each item of the BNT and resulted in α = .41.

#### Arithmetic calculation

Arithmetic calculation ability was assessed using a similar procedure as Gebuis and van der Smagt [57] and Lindskog et al. [10]. This test was divided into four subtests (addition, subtraction, multiplication, and division). For each subtest, the participants were faced with a sheet of paper containing printed arithmetic problems of increasing difficulty. For each subtest, they were instructed to complete as many problems as they could within the allotted time of 120 seconds. A brief pause was included in between each subtest. The difficulty level of the problems was manipulated by increasing the number of digits or by requiring borrowing or carrying. Each subtest contained 54 problems to be solved. The total number of correctly solved arithmetic problems across all four conditions was used as a measure of arithmetic calculation ability. Cronbach’s alpha was calculated by checking the internal consistency across each arithmetic subtest and resulted in α = .88.

### Procedure

The testing was divided into two separate sessions, mainly to avoid fatigue and carryover effects of sensitive tasks (for example, the math anxiety questionnaire and measures of math ability were completed in separate sessions). In the first session, the participants completed the numeracy test and the arithmetic calculation test and other self-report questionnaires concerning various demographical variables not reported here. In the second session, the participants completed the math anxiety questionnaire, the WAIS-IV digit subtest, and the symbolic number discrimination task. All testing was completed within one month. Instructions were read aloud by an experimenter from a printed manuscript and all tests were administered in the same order for all study participants. Computer-based tasks were run on a laptop, using SuperLab PRO 4.5.

## Results

An overview of the descriptive results can be found in Table 1 below. The Mplus 7 software [58] was used to estimate the models.

**Table 1:** Overview of descriptive data from the measured variables.

	1	2	3	4	5	6	7	8	9	10	11	Mean (SD)
1. Gender^α^	0.25											0.50 (0.50)
2. MA (MEA)	–.34**	36.94										19.95 (6.08)
3. MA (ESA)	–.08	.49**	11.81									12.38 (3.44)
4. MA (MOA)	–.10	.56**	.27**	6.05								7.89 (2.46)
5. WM (DSF)	.15*	–.18*	–.02	–.13	3.02							9.27 (1.74)
6. WM (DSB)	.02	–.23*	–.13	–.20*	.46**	4.13						9.00 (2.03)
7. WM (DSS)	.14	–.28**	–.13	–.15*	.48**	.43**	3.79					9.42 (1.95)
8. NP (1-DC)	–.20*	.29**	.07	.31**	–.21*	–.16*	–.23*	9080.48				540.06 (95.29)
9. NP (2-DC)	–.15*	.18*	.06	.20*	–.25**	–.21*	–.26**	.73**	14242.60			789.06 (119.34)
10. Numeracy	.29**	–.35**	–.05	–.17*	.25**	.38**	.31**	–.16*	–.13	1.48		2.18 (1.22)
11. Arithmetic	.31**	–.52**	–.15	-.26**	.40**	.40**	.45**	–.43**	–.48**	.50**	507.54	106.29 (22.53)

Correlations are presented under the diagonal, variances in the diagonal and mean and standard deviation are to the right.

* = Statistically significant (*p* < .05),

** = Statistically significant (*p* < .001),

^α^ = Females are coded as 0 and males as 1.

### Path analysis of math anxiety and arithmetic calculation

**Fig 2:**
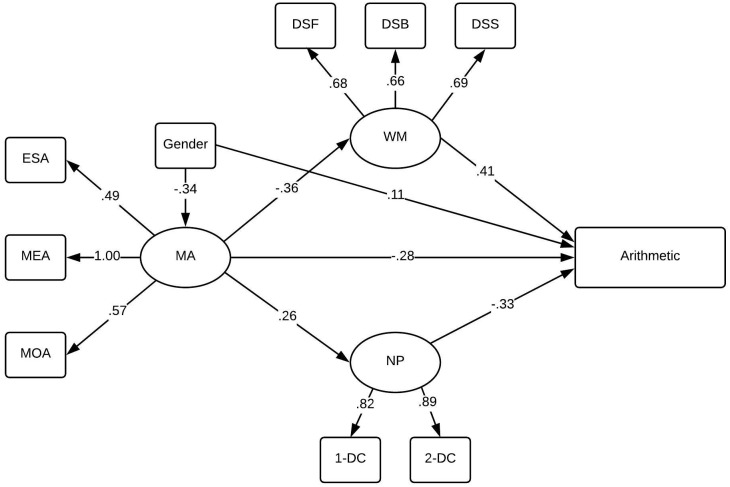
Path analysis of arithmetic and math anxiety. The relation between number processing (NP), math anxiety (MA) working memory (WM) and arithmetic ability. All paths were at *p <* .*05*.

We tested our hypothesized conceptual model in a confirmatory approach, and the resulting model can be found in Fig 2 below. Due to a negative residual variance of the maturity indicator the residual variance was set to 0.01 in the model. The model showed reasonable fit, χ^2^ = 49.14 (31), *p* = . 020, CFI = .96, RMSEA = 0.06, C. I (90%) = 0.02–0.09. The path from math anxiety via working memory had a statistically significant indirect effect to arithmetic calculation (*p* = .001, standardized estimate = -.15) and also a significant indirect effect via NP to arithmetic (*p* = .027, standardized estimate = -.09). The model explained 56% of the variance of arithmetic.

### Path analysis of math anxiety and numeracy

The model showed good fit, χ^2^ = 44.57 (32), *p* = . 069, CFI = .97, RMSEA = 0.05, C. I (90%) = 0.00–0.08. Due to a negative residual variance of the MEA indicator and 1-DC indicator they were both set to 0.01 in the model. The path from math anxiety via working memory had a statistically significant indirect effect to numeracy (*p* = .004, standardized estimate = -.14) but not a significant indirect effect via NP to numeracy (*p* = .807). The model explained 29% of the variance in numeracy.

## Discussion

The focus of the current study was to contribute to our understanding of the mechanisms by which MA undermines mathematical abilities in adults. It has since long been established that MA is related to poor math performance, and indirectly to education and career path choice [11] [43] [44], and that MA affects performance long into adulthood. Still, the mechanisms by which it affects math abilities remain elusive. Using SEM we could determine plausible pathways through which it acts on math performance. Specifically, we juxtaposed two different accounts of how MA interferes with mathematical processing. The first account maintains that individuals with MA are inflicted with negative emotions that prompts emotional and cognitive control responses that in turn drain WM resources available for the task at hand (e.g., [11] [43]). According to this account, MA should affect math ability indirectly through WM. According to the second account, MA primarily interferes with mathematics abilities through poorer basic number processing [51] [12]. Therefore, MA should indirectly influence math ability through basic number processing ability. Given that different cognitive abilities support different aspects of mathematics [16] [17] [18] [19] we also investigated whether MA undermines two different aspects of math to the same degree and through the same pathways.

**Fig 3:**
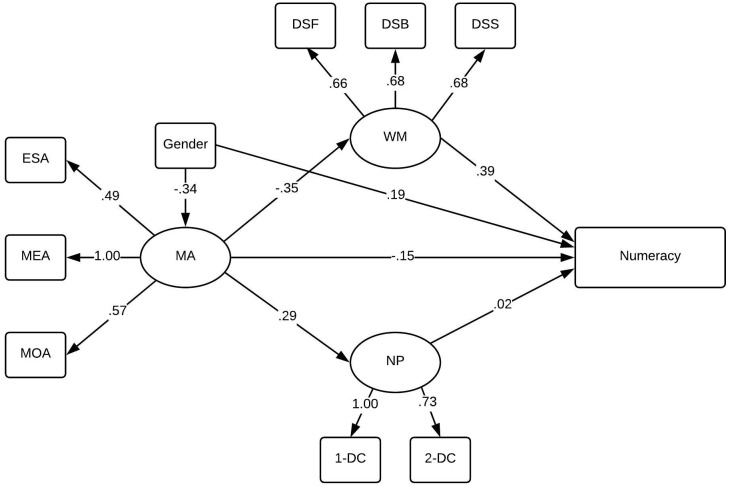
Path analysis of numeracy and math anxiety. The relation between number processing (NP), math anxiety (MA) working memory (WM) and numeracy. All paths were at *p <* .*05*.

In terms of the results, and as can be seen in Figs 2 and 3, the overall pattern is similar for both arithmetic calculation ability and numeracy. As expected, we replicated previous research establishing strong links between WM, numeracy and arithmetic ability (e.g., [20]). Similarly, we replicate findings that have established links between symbolic number processing and arithmetic and numeracy in adults [41]. Symbolic number processing has consistently been associated with mathematics ability in older children and adults (e.g., [41] [59] [42] [60]). We also find support for the notion that basic number processing is more important for fundamental aspects of math, such as arithmetic, as opposed to more sophisticated mathematics, such as probability estimates in the case of numeracy. This is also shown in a meta-analysis by Schneider et al. [60] who illustrated that the association between basic number processing and mathematics is higher for aspects related to early mathematics (e.g., arithmetic) than for more sophisticated aspects more prevalent in later mathematics curricula. A similar argument maintains that basic number processing skills play an important role in aspects of mathematics that are tightly linked to the whole number system, such as multi-digit calculation [18]. On the other hand, the numeracy problems are demanding in terms of the problem structure, requiring multistep calculations and abstract reasoning, which explains why performance on the numeracy measure was more influenced by WM. In addition, WM processes allows for temporary storage of intermediate results of the calculations that are involved in solving the problem. Verbal WM has been tied to performance at word problem solving [61], and given the nature of the rather language infused numeracy test, the results are congenial with Fuchs et al. [61]. Still, we show that basic number processing skills continues to be important for adult mathematics ability even while including WM capacity in the model.

By including gender as a variable in our models, we also corroborate prior research demonstrating an effect of gender on MA [52] and math abilities. The gender effect has been suggested to be more pronounced as a function of age and may be driven by gender stereotypes or transmission of anxiety by female teachers who themselves are anxious about math [62].

Our findings indicate that MA may influence math ability through three distinct pathways in both types of mathematics. Thus, contrary to the initial juxtaposition of the two differential pathways through which MA has been suggested to operate, the models indicate that MA show a combined effect. For arithmetic calculation ability, we find that MA has an indirect effect through both WM and basic number processing as well as a direct effect above and beyond the indirect effects. Still, the effect on numeracy was largely attributed to the pathway between MA and WM, which supports the notion that the primary driver behind the performance decrement in mathematics processing is derived from an interference of general cognitive processes–the affective drop. Although we could establish a link between MA and symbolic number processing, this relationship had a much smaller effect on numeracy compared to the pathway between MA and WM, and MA had no significant indirect effect through the number processing pathway. These models give credence to the notion that individuals with MA may primarily suffer from an affective drop in performance during processing of numerical stimuli. This is in line with neuroimaging studies of MA in which likely mechanisms can be traced to aberrant activity of the amygdala [50] and inefficient deregulation of the DMN in the brain [46]. Beyond the simple juxtaposition between the two different accounts of how MA affect math ability (i.e. number processing vs. WM), we also show that MA relates to math ability through a direct pathway. This remaining effect may be derived from more distal mechanisms, such as prolonged avoidance behavior of math courses and engagement in activities reliant upon processing of numbers and math. Thus, not only does MA proximally influence cognitive processing *in situ*, but also distally by keeping individuals from honing their mathematical skills and their comfort with numbers. This line of reasoning was also raised by Douglas and Lefevre [52] who suggested that experiential effects, such as avoidance behavior, may affect math learning in conjunction with direct cognitive processes. This interpretation could be investigated by testing younger children using the same paradigm as in the current study. If the direct pathway from MA to math ability can be attributed to avoidance effects, the effect should be weaker or nonexistent in children.

Our results differ somewhat from the results reported by Douglas and Lefevre [52] despite having a similar study design. The authors found no direct link between basic number processing or cognitive abilities and MA. One important difference resides in the fact that they used MA as the primary outcome variable and investigated the putative causes of MA, as opposed to the current study in which we investigated how MA ultimately impair mathematical performance. Given the likely bidirectional relationship between MA and math performance that may influence one another in a vicious cycle, both approaches are valid and necessary in order to disentangle this intricate relationship. However, both approaches need to be complemented by independent longitudinal and experimental manipulations in order to make firm claims about the directions of causality.

Summing up the results, we reconcile previous reports about the putative mechanisms by which MA interferes with mathematical processing. A novel contribution is that we have successfully modeled the role of MA in mathematics performance using SEM that confirm the multifaceted role of MA, which go against the notion that MA undermines mathematics through a singular mechanism. In addition, we also find that the relative impact of MA and the pathway with which is affects mathematics varies depending on what aspect of math is being considered. During pure arithmetic calculation, MA works through both WM and basic number processing, whereas more abstract mathematical reasoning (i.e., numeracy) is to a greater degree linked to WM resources.

What are the ramifications of these patterns of results and what can be done to ameliorate these adverse effects? One possible way in which one might be able to leverage the current results would be to tailor interventions directed at each specific pathway. For example, concerning the role of WM, interesting intervention studies have already been tried with promising results. Studies have found that expressive writing, or “writing out” negative affect and worry, prior to exams and other math situations lowered ruminating behavior and thus mitigated the negative impact on WM resources which in turn had a positive influence on mathematical performance [63] [64]. In terms of the second pathway through basic number processing, one potential course of action would be to employ a kind of exposure-based therapy in which individuals are exposed to numerical information and thus become desensitized to numerical symbols. In this respect, a promising approach was offered by Supekar and colleagues [65] who found that children with high MA showed lower amygdala activity after a period of intensive math tutoring program. To address the third pathway through which MA hampers mathematical performance, one would likely have to start early and target the sociocultural climate in which the child is embedded. For instance, it could very well be the case that the negative and anxious attitudes towards math has been transmitted from either parents or teachers who themselves dislike mathematics [66] or that initial failure in mathematics in primary school sparks a negative and vicious spiral of avoidance behavior and subsequent failures as a result. This is a complex issue and would require a combined effort of schools and parents. Still, it is without a question a desirable outcome to try to foster a positive learning environment both at home and in school to instill a positive attitude towards mathematics. In a best case scenario, interventions are put into action early on in primary school, fostering a positive attitude towards math and numbers, after which these interventions may show compound effects in downstream mathematics abilities and behavior, thus reducing the need to initiate desensitization protocols in adolescents and adults later on.

### Limitations

A major limitation of the current study was the choice to use the BNT rather than a more comprehensive math achievement test, such as the Woodcock-Johnson III Tests of Achievement subtests [67]. Although the BNT has shown valid psychometric properties [20] and was correlated with the arithmetic calculation measure in the current data (*r* = .50, *p <* .001), the limited number of items provides a statistical limitation. The reliability coefficient was calculated at α = .41, which is very low. Despite being carefully crafted, this is a byproduct of how the BNT was created. The creators of the BNT carefully chose items such that each question reliably could discriminate a quartile in a normally distributed way in the population [20] [56]. Together with the fact that it is a short test, it will inevitably show a low internal reliability. Nevertheless, others have reported a test-retest reliability coefficient of .91 [20]. However, this still provides a statistical limitation regarding how well the SEM models can be fitted to the data. As such, the conclusions relying on the BNT should be treated with caution until future studies can reinforce the claims as a form of convergent validity. Thus, future studies should investigate how other measures of math ability relate to the measures used in the current study. Even though we have successfully modeled how MA impair mathematics abilities, much remains to be done in terms of enhancing our understanding of MA. For example, it is absolutely imperative that we use longitudinal approaches to investigate the long-term trajectory of MA and the relationship to cognitive abilities and processes. Different mechanisms may be pronounced at different stages in ontogeny. The correlational nature of the current study thus require a longitudinal and experimental paradigm to corroborate the models provided herein. Also, studies utilizing longitudinal and experimental manipulations are warranted to make firm claims about the directions of causality. The intricate relationship between neural mechanisms, cognitive mechanisms, and social mechanisms provide a hefty challenge to untangle. Still, researchers in different disciplines have begun to tackle this important challenge, making for a promising outlook at its potential resolution.

### Conclusion

Taken together, our findings using SEM indicate that MA may impede math performance through three pathways: (1) indirectly through working memory ability, giving support for the ‘affective drop’ hypothesis of MA’s role in mathematical performance, (2) indirectly through basic number processing, corroborating the notion of domain-specific mechanisms pertaining to number, and (3) a direct effect of MA on math performance, possibly due to distal avoidance behavior. Thus, we reconcile different accounts of how MA may affect mathematics. Importantly, the pathways vary in terms of their relative strength depending on what type of mathematical problems are being solved. These findings shed light on the mechanisms by which MA interferes with mathematical performance by highlighting the multifaceted role of how MA affects math performance both proximally and distally.

## Supporting information

S1 File Dataset. Dataset including variables on which the current study is based.
(ZIP)
